# Supplementary material for: Identification of photoperiod-induced specific miRNAs in the adrenal glands of Sunite sheep (Ovis aries)
Source: Front Vet Sci. 2022 Jul 22;9:888207. doi: 10.3389/fvets.2022.888207 (PMC9354845; doi:10.3389/fvets.2022.888207)
Supplement: Supplementary file 1 [file Data_Sheet_1.ZIP › Supplementary file/miRDeep2 file.zip!/miRDeep2 file/Novel_Structure/LP42A3/Novel_516.pdf]

Provisional ID	:	NC_019483.2_23953
Score total	:	19.2
Score for star read(s)	:	3.9
Score for read counts	:	11.9
Score for mfe	:	1.9
Score for randfold	:	1.6
Score for cons. seed	:	
Total read count	:	35
Mature read count	:	33
Loop read count	:	0
Star read count	:	2

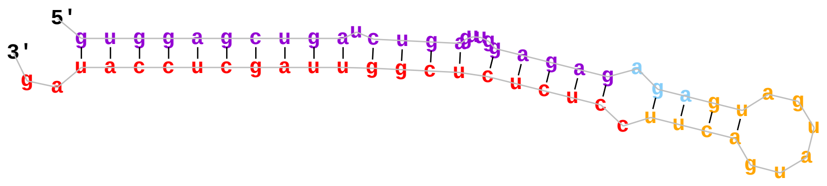

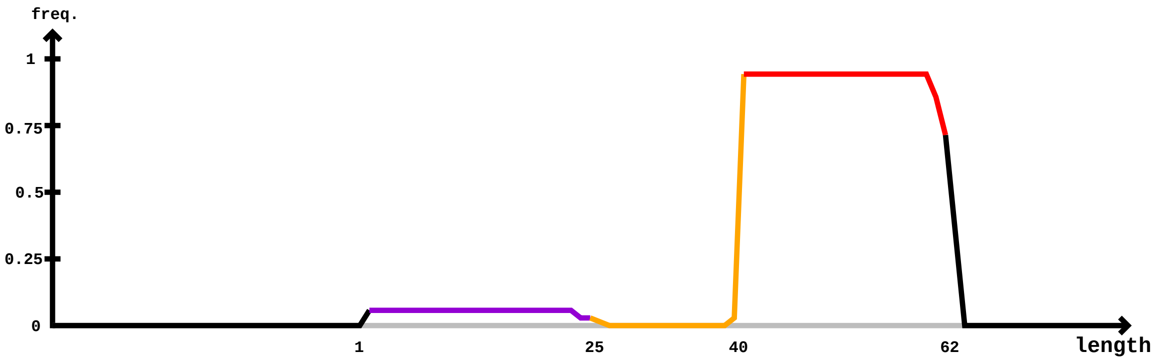


```
                                  Star                                          Mature
5'- uuuacucaguucacugaccgauugugccaguguggagcugaucugaguuggagagagaguaguaugacuuccucucucgguuagcuccauagauggcagaucagccuucu  -3'  obs
    uuuacucaguucacugaccgauugugccaguguggagcugaucugaguuggagagagaguaguaugacuuccucucucgguuagcuccauagauggcagaucagccuucu       exp
    .....((((....))))..((((.(((((.(((((((((((.((((....(((((.((((......)))).))))))))))))))))))))..)))))))))........   reads   mm   sample
    ...............................guggagcugaucugaguuggag.........................................................   1       0    seq
    ...............................guggagcugaucugaguuggagag.......................................................   1       0    seq
    .....................................................................uccucucucgguuagcuccauag..................   1       0    seq
    ......................................................................ccucucucgguuagcuccau....................   3       0    seq
    ......................................................................ccucucucgguuagcuccaua...................   5       0    seq
    ......................................................................ccucucucgguuagcuccauag..................   19      0    seq
    ......................................................................ccucucucgguuagcuccauaga.................   5       0    seq
```
